# Cardiopulmonary, Functional, Cognitive and Mental Health Outcomes Post-COVID-19, Across the Range of Severity of Acute Illness, in a Physically Active, Working-Age Population

**DOI:** 10.1186/s40798-023-00552-0

**Published:** 2023-02-02

**Authors:** Oliver O’Sullivan, David A. Holdsworth, Peter Ladlow, Robert M. Barker-Davies, Rebecca Chamley, Andrew Houston, Samantha May, Dominic Dewson, Daniel Mills, Kayleigh Pierce, James Mitchell, Cheng Xie, Edward Sellon, Jon Naylor, Joseph Mulae, Mark Cranley, Nick P. Talbot, Oliver J. Rider, Edward D. Nicol, Alexander N. Bennett

**Affiliations:** 1 Academic Department of Military Rehabilitation (ADMR), Defence Medical Rehabilitation Centre (DMRC) Stanford Hall, Loughborough, LE12 5QW UK; 2 Academic Unit of Injury, Recovery and Inflammation Sciences, University of Nottingham, Nottingham, UK; 3 Academic Department of Military Medicine, Birmingham, UK; 4 Oxford University Hospitals NHS Foundation Trust, Oxford, UK; 5 Department for Health, University of Bath, Bath, UK; 6 School of Sport, Exercise and Health Sciences, Loughborough University, Loughborough, UK; 7 Royal Centre for Defence Medicine, Birmingham, UK; 8 Metabolic Neurology, Institute of Metabolism and Systems Research, University of Birmingham, Birmingham, UK; 9 Defence Medical Rehabilitation Centre (DMRC), Stanford Hall, Loughborough, UK; 10 Department of Physiology, Anatomy and Genetics, University of Oxford, Oxford, UK; 11 University of Oxford Centre for Clinical Magnetic Resonance Research, University of Oxford, Oxford, UK; 12 Department of Cardiology, Oxford University Hospitals NHS Foundation Trust, Oxford, UK; 13 Royal Brompton Hospital, London, UK; 14 National Heart and Lung Institute, Imperial College London, London, UK

**Keywords:** Coronavirus disease 2019, Long Covid, Post-COVID-19 syndrome, Cardiopulmonary exercise testing, Outcomes

## Abstract

**Background:**

The COVID-19 pandemic has led to significant morbidity and mortality, with the former impacting and limiting individuals requiring high physical fitness, including sportspeople and emergency services.

**Methods:**

Observational cohort study of 4 groups: hospitalised, community illness with on-going symptoms (community-symptomatic), community illness now recovered (community-recovered) and comparison. A total of 113 participants (aged 39 ± 9, 86% male) were recruited: hospitalised (*n* = 35), community-symptomatic (*n* = 34), community-recovered (*n* = 18) and comparison (*n* = 26), approximately five months following acute illness. Participant outcome measures included cardiopulmonary imaging, submaximal and maximal exercise testing, pulmonary function, cognitive assessment, blood tests and questionnaires on mental health and function.

**Results:**

Hospitalised and community-symptomatic groups were older (43 ± 9 and 37 ± 10, *P* = 0.003), with a higher body mass index (31 ± 4 and 29 ± 4, *P* < 0.001), and had worse mental health (anxiety, depression and post-traumatic stress), fatigue and quality of life scores. Hospitalised and community-symptomatic participants performed less well on sub-maximal and maximal exercise testing. Hospitalised individuals had impaired ventilatory efficiency (higher VE/V̇CO_2_ slope, 29.6 ± 5.1, *P* < 0.001), achieved less work at anaerobic threshold (70 ± 15, *P* < 0.001) and peak (231 ± 35, *P* < 0.001), and had a reduced forced vital capacity (4.7 ± 0.9, *P* = 0.004). Clinically significant abnormal cardiopulmonary imaging findings were present in 6% of hospitalised participants. Community-recovered individuals had no significant differences in outcomes to the comparison group.

**Conclusion:**

Symptomatically recovered individuals who suffered mild-moderate acute COVID-19 do not differ from an age-, sex- and job-role-matched comparison population five months post-illness. Individuals who were hospitalised or continue to suffer symptoms may require a specific comprehensive assessment prior to return to full physical activity.

**Supplementary Information:**

The online version contains supplementary material available at 10.1186/s40798-023-00552-0.

## Key Points

- This study demonstrates that, in a physically active, working-age population, those who are symptomatically recovered from mild-moderate COVID-19 do not differ in any parameter from a comparison group of uninfected individuals matched for age, sex and job-role.
- Those who were hospitalised and community-managed patients with ongoing symptoms have worse outcomes in terms of cardiopulmonary imaging findings, functional capacity and mental health status compared to both community-recovered and comparison groups.
- Individuals whose occupation or recreation requires high intensity physical activity, who have either had severe disease requiring hospitalisation, or are suffering persistent symptoms beyond 12 weeks, may require specific, focussed assessment prior to a return to full physical activity.

## Introduction

Severe acute respiratory syndrome coronavirus-2 (SARS-CoV-2), and resulting coronavirus disease 2019 (COVID-19), continues to cause significant mortality and morbidity, with over 620 million confirmed cases, and 6.5 million deaths globally [1]. Approximately 80% of SARS-CoV-2 cases are asymptomatic or mild, with many patients recovering within 2–4 weeks [2]. However, COVID-19 also causes prolonged illness, with some individuals experiencing persistent symptoms for months, including shortness of breath (SoB), fatigue and mood disturbance [3–7. The National Institute for Health and Care Excellence (NICE) have adopted time-based definitions for post-COVID illness: after four weeks, ‘ongoing symptomatic COVID-19’, and beyond 12 weeks, ‘post-COVID-19 syndrome’ [8]. An estimated 2.3 million people in the UK (population: 66 million) have ongoing symptoms at ≥ 4 weeks [9].

The mean age of post-COVID-19 syndrome sufferers is ~ 40 years, whilst approximately 20% of previously healthy 18–35 year olds report ongoing symptoms at 14–21 days, implying the majority of negatively affected individuals are in the working population [10, 11]. This has consequences for return to work and economic recovery. Initial studies found the severity and duration of acute COVID-19 increased the risk of chronicity, but this is now challenged [12, 13]. Most studies investigating post-COVID-19 syndrome have focussed on those hospitalised with COVID-19, not those who remained in the community, and only a few utilise a control population [3, 5, 14–23]. Ongoing symptoms consistently include SoB, fatigue, pain, mood disorders and perceived cognitive impairment [3, 15]. Cross-sectional cardiopulmonary imaging abnormalities, including lung fibrosis and myocardial inflammation, [24, 25] and functional limitations have been recorded [26–28].

An inability to fully recover from COVID-19 has a high impact on populations who require a high level of physical fitness and decision-making, such as professional athletes and front-line emergency services (e.g. police, firefighters, paramedics, military). These populations are exposed to high volume and/or intensity exercise, often under challenging environmental conditions, and enduring pathology would impair their return to high-end physical and cognitive function in high-pressure situations.

Alongside a specifically commissioned clinical service [29], the Military COVID-19 Observational Outcomes in a Viral Infectious Disease (M-COVID) study was developed to describe the longitudinal effects of SARS-CoV-2 on the UK Armed Forces in three groups: hospitalised illness (H), community illness with on-going symptoms (community-symptomatic, CS) and community illness now recovered (community-recovered, CR).

This study aims to describe cardiopulmonary, functional, and neurocognitive outcomes five months post-illness, comparing the post-COVID-19 groups with each other and an age-, gender- and job-role-matched comparison group (COM), with the hypothesis that those with more severe initial or prolonged disease have worse outcomes.

## Methods

### Study Design

MCOVID is a cross-sectional observational cohort study, five months post-acute illness. Ethical approval was granted by the Ministry of Defence research ethic committee in July 2020 (1061/MODREC/20).

### Patient and Public Involvement

Multiple focus groups were held at the Defence Medical Rehabilitation Centre (DMRC) Stanford Hall with potential participants during the study design phase (June and July 2020). Iterative feedback was gained on the patient information leaflet, study concept and design, and study visit details.

### Setting and Study Overview

**Fig. 1:**
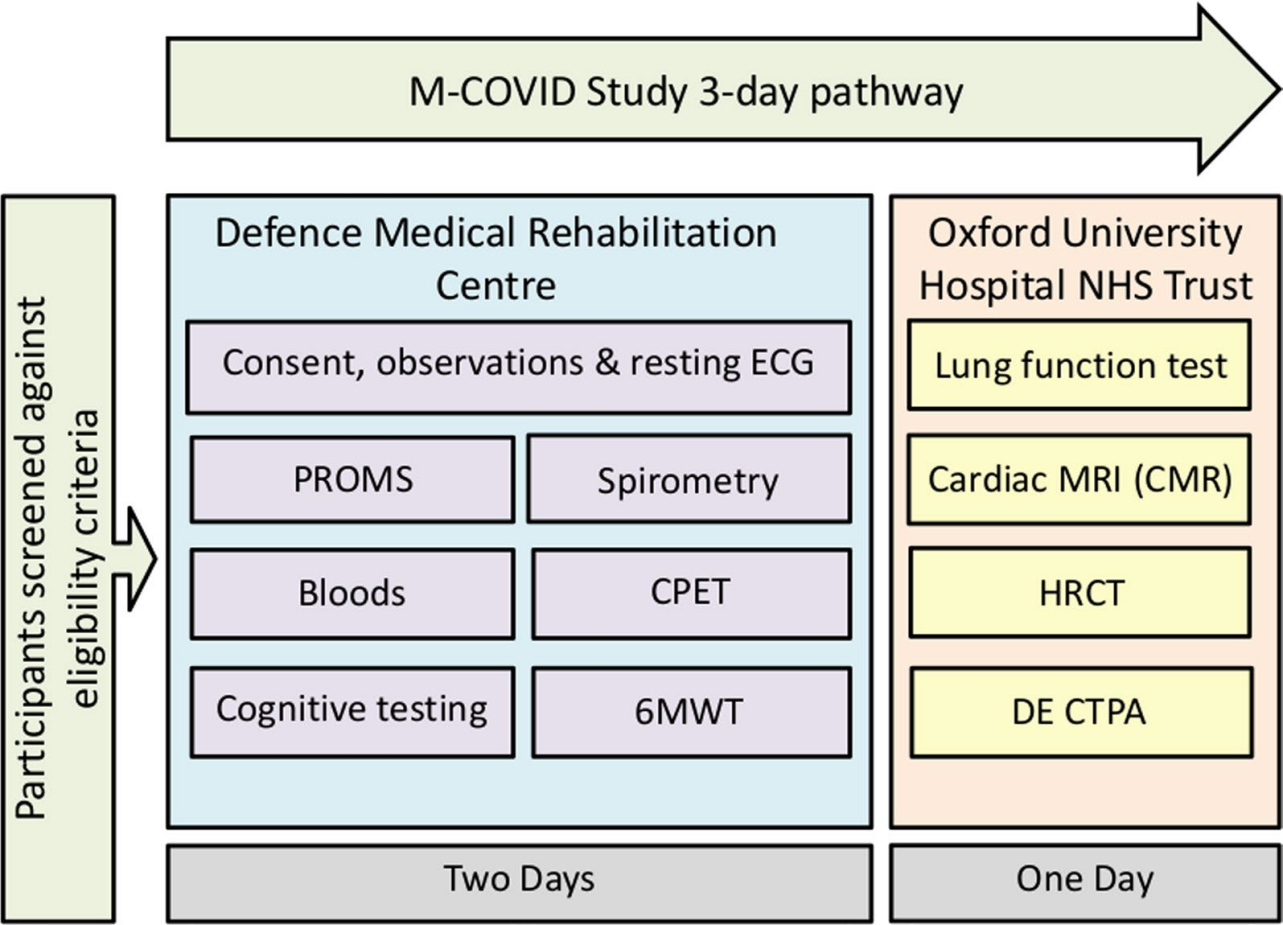
Diagrammatic description of study design. Abbreviations: ECG, electrocardiogram; PROMS, patient-reported outcome measure; CPET, cardiopulmonary exercise test; 6MWT, six-minute walk test; MRI, magnetic resonance imaging; CMR, cardiac magnetic resonance imaging; HRCT, high-resolution computed tomography; DE CTPA, dual-energy computed tomography pulmonary angiogram

Initial visits occurred over three days between August 2020 and July 2021. There were two days at DMRC for cardiopulmonary exercise testing (CPET), 6-min walk test (6MWT), cognitive assessment, spirometry, blood samples and patient-reported outcome measures (PROMs) and a third at Oxford University Hospital (OUH) NHS Foundation Trust for cardiopulmonary imaging and additional pulmonary function testing (Fig. 1).

### Participants

**Fig. 2:**
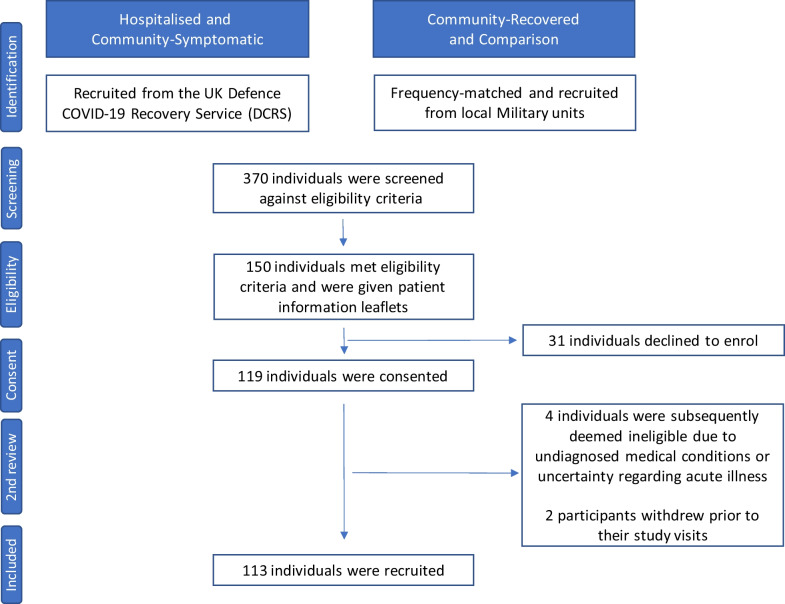
CONSORT flow diagram of patient recruitment

A total of 370 participants were screened, with 150 approached and 119 consented (Fig. 2). Two consultants adjudicated consenting volunteers meeting eligibility criteria (Table 1) based on positive SARS-CoV-2 antigen, history, blood tests and imaging, excluding four for previously undiagnosed medical conditions. Two participants withdrew mid-study visit.

**Table 1:** Inclusion and exclusion criteria for the Military COVID-19 Observational Outcome in a Viral Infectious Disease (M-COVID) study

Inclusion criteria				Exclusion criteria
Hospitalised	Community-symptomatic	Community-recovered	Comparison	All groups
Currently serving UK Military Personnel at time of recruitment	Currently serving UK Military Personnel at time of recruitment	Currently serving UK Military Personnel at time of recruitment	Currently serving UK Military Personnel at time of recruitment	Unwilling or unable to give informed consent
Previous COVID-19 infection—requiring hospital admission	Previous COVID-19 infection—self-limiting mild/moderate acute illness, not requiring hospital admission	Previous COVID-19 infection- self-limiting mild/moderate acute illness, not requiring hospital admission	No previous clinical suspicion of acute COVID-19 infection	Past medical history of cardiac or pulmonary disease (excluding mild asthma and controlled hypertension)
Positive COVID-19 antigen PCR test at time of acute illness, or clinically adjudicated COVID-19	Positive COVID-19 antigen PCR test at time of acute illness, or clinically adjudicated COVID-19	Positive COVID-19 antigen PCR test at time of acute illness, or clinically adjudicated COVID-19	Negative COVID-19 antibody test at recruitment to study	Active acute infection at the time of recruitment as determined by clinical assessment
Negative for COVID-19 antigen PCR test at recruitment to study	Negative for COVID-19 antigen PCR test at recruitment to study	Negative for COVID-19 antigen PCR test at recruitment to study	Negative for COVID-19 antigen PCR test at recruitment to study	Unable to meet the criteria of the MRI safety questionnaire
> 4 weeks since resolution of fever/acute illness	> 4 weeks since resolution of fever/acute illness	> 4 weeks since resolution of fever/acute illness	–	Pregnant at the time of recruitment to the study
–	Ongoing post-acute symptoms/exercise intolerance	No ongoing post-acute symptoms/exercise intolerance	–	Unable, or unwilling, to complete any research investigations

Abbreviations: COVID-19, Coronavirus disease 2019; PCR, polymerase chain reaction; MRI, magnetic resonance imaging

A total of 113 participants were categorised into 1 of 4 cohorts; hospitalised (*n* = 35); community-symptomatic (*n* = 34); community-recovered (*n* = 18) and comparison (*n* = 26). Exposed participants were recruited via the clinical pathway established in August 2020 for those with initially severe or prolonged COVID-19 illness to ensure safe return to duty [29].

Hospitalisation during acute illness was used pragmatically as a marker of severity. All hospitalised participants required supplementary oxygen. Recovered and comparison participants (frequency-matched by the study team to age, gender and job-roles) were identified and recruited using word-of-mouth. All comparisons were SARS-CoV-2 nucleocapsid antibody negative (positive if prior illness).

### Determining Recovery Status

Non-recovery was defined as the continued presence of one or more of the below post-COVID-19 symptoms at recruitment (Table 2).

**Table 2:** Prevalence of symptoms across all groups

Symptom	H (%)	CS (%)	CR (%)	COM (%)
Any shortness of breath	63	71	0	0
Fatigue	54	68	0	4
Chest pain	20	35	0	0
Exercise intolerance	20	35	0	0
Joint pain	26	15	0	0
Loss of smell	9	21	0	0

Abbreviations: H, hospitalised illness; CS, community illness with on-going symptoms (community-symptomatic); CR, community illness now recovered (community-recovered); COM, age-, gender- and job-role-matched comparison population

### Variables

#### Job Role and Rank

Participant job role was recorded, to ensure that those in Ground Close Combat roles (subject to higher physical activity standards) had appropriate matched comparators. Rank was used as a proxy for socioeconomic status (SES) [30, 31].

#### Baseline Observations

Heart rate (HR), blood pressure (BP), temperature and peripheral oxyhaemoglobin saturations (SpO_2_) were acquired by an IPM 8 Mindray Patient Monitor (Mindray UK Ltd, Huntingdon, UK).

#### Venous Blood Sampling

Samples for full blood count, liver function, urea and electrolytes, C-reactive protein, creatine kinase, thyroid function, ferritin and iron studies, vitamin D, and COVID-19 antibodies (spike and nucleocapsid) were taken.

### Cardiopulmonary Functional Testing

#### Six-Minute Walk Test (6MWT)

6MWTs were performed using standardised guidelines [32], with pre-test body composition recorded (stature, body mass, hip and waist circumference). A pulse oximeter (Nonin Onyx Vantage 9590, Minnesota, USA) was used to measure HR and SpO_2_, with participant’s rate of perceived exertion (RPE, 6–20) [33] and SoB (0–10) [34] recorded, pre- and post-test.

#### Cardiopulmonary Exercise Testing (CPET)

CPET was conducted on an electromagnetically braked cycle ergometer (Lode Carnival, Lobe BV, Groningen, Netherlands) using indirect calorimetry (Metalyzer 3B, Cortex Biophysik, Leipzig, Germany) with continuous 12-lead ECG monitoring (Custo Diagnostic software, Custo-Med, Ottoburn, Germany). A ramp protocol to volitional fatigue was employed, with a maximal test that was defined by a respiratory exchange ratio, RER, of > 1.1 and a plateau in V̇O2 over 30-s despite increasing workload [36]. The protocol started with a two-minute rest period, then two-minutes of unloaded pedalling, followed by progressive increase in workload based on a workload/min ramp to achieve 8–12 min of loaded exercise.

Ventilation (V̇E), oxygen consumption (V̇O2), expired carbon dioxide (V̇CO2), HR and SpO2 were monitored continuously [36], with BP, RPE and perceived SoB recorded every two minutes.

#### Spirometry and Pulmonary Function Test

Standing spirometry assessments (MicroMedical MicroLab 3500, CA, USA) were taken to measure forced vital capacity (FVC) and forced expiratory volume in the first second of expiration (FEV1) [35]. The diffusing capacity of the lungs for carbon monoxide (DLCO) was measured over a 10-s breath hold, using methane as a tracer gas.

### Cardiopulmonary Imaging

#### Cardiothoracic Imaging

High-resolution computed tomography (HRCT) chest and dual-energy CT pulmonary angiography (DECTPA) were performed on a dual-source CT (Siemens SOMATOM Drive, Siemens Healthineers, Erlangen, Germany), using a HRCT protocol of inspiratory 1 mm sections with 10 mm gap, and expiratory 1 mm sections with a 30-mm gap. DECTPA perfusion map and reconstructed 1 mm slice thickness were analysed on Siemens Syngo, CT CE Lung Analysis software. Comparison participants did not undergo CT imaging.

#### Cardiac Magnetic Resonance Imaging (CMR)

CMRs were acquired on Siemens MR scanners at 3 Tesla (Siemen Medical Solutions, Erlangen, Germany), assessing myocardial mass, volumes and ejection fraction with precordial ECG gating, in held end-expiration. Mapping sequences (ShMOLLI, Siemens) and late gadolinium imaging were obtained with a bolus injection of 0.1 mmol/kg of a gadolinium contrast agent. Images were analysed with CVI 42 analysis software (Circle Cardiovascular Imaging Inc, Calgary, AB, Canada).

### Patient-Reported Outcome Measures

Participants completed PROMs relating to depression (Patient Health Questionnaire-9, PHQ-9) [38]; anxiety (General Anxiety Disorder scale-7 questions, GAD-7) [39]; post-traumatic stress disorder (PTSD, National Centre for PTSD checklist, PCL-5) [40]; quality of life (QoL, European QoL 5 domains,EQ5D) [41], and fatigue (Fatigue Assessment Scale, FAS) [42]. Ongoing symptoms were measured using an evidence-based symptom checklist [43, 44].

### Cognitive Assessment

Cognitive assessments were performed using the National Institute of Health (NIH) Cognitive Toolbox cognition battery for age 12+ years on an iPad (Apple, California, USA) [37], with the fluid, crystallised and total composite scores analysed. Highest educational level was recorded during this and also used as a proxy for SES [30].

### Data Management and Statistical Methods

Study data were collected and managed using REDCap [45].

### Statistical Analysis

Data are presented as mean ± standard deviation. The normality of all variables was assessed using a Shapiro–Wilk test and inspection of the frequency histogram distributions and Q–Q plots. Results showed approximate normal distribution across the majority of variables, except the PROMs, namely GAD-7, PHQ-9, PCL-5, EQ5D and FAS. Parametric tests were applied for all variables except PROMs, when nonparametric tests were applied.

To measure for differences in demographics, functional, neurocognitive and mental health status, and cardiopulmonary function/pathology between the four groups, a one-way analysis of variance (ANOVA) was performed on all continuous data and a Chi-squared test on ordinal and categorical data, where the groups were used as the columns and the independent variable as the rows for the Chi-squared analysis. To measure for differences in the neurocognitive and mental health status between the four groups, Kruskal–Wallis tests were applied.

An alpha threshold of 0.05 was taken to indicate significance. Post hoc tests were carried out for any results where a significant between-group difference was identified following an ANOVA. Bonferroni corrections were applied to allow for multiple post hoc comparisons.

## Results

At review (159 ± 72 days following acute illness), hospitalised and community-symptomatic individuals had a mean of 2 ± 2 and 2 ± 1 symptoms, respectively (Table 2). Hospitalised individuals were significantly older than both community-symptomatic and community-recovered (Table 3).

**Table 3:** Descriptive data demonstrating body composition, ambulatory function, mental health and fatigue status

	H	CS	CR	COM	*F* Value	*P* value	Post hoc comparison
Number	35	34	18	26			
Age	43 ± 9	37 ± 10	34 ± 6	38 ± 8	4.856	**0.003**	¶*, †**
Time to assessment	145 ± 63	166 ± 65	142 ± 53	–	0.910	**0.408**	
Body composition							
Height (cm)	176 ± 7	179 ± 10	180 ± 8	176 ± 8	1.157	0.330	
Body mass (kg)	96 ± 15	94 ± 19	83 ± 11	79 ± 8	10.083	** < 0.001**	†*, §***, ¥***
Body mass index (kg m^2^)	31 ± 4	29 ± 4	26 ± 2	25 ± 3	17.909	** < 0.001**	†***, §***, #**, ¥***
Waist circumference	101 ± 13	96 ± 13	85 ± 10	86 ± 7	13.923	** < 0.001**	†***, §***, #**, ¥**
Waist-to-hip ratio	0.96 ± 0.09	0.94 ± 0.12	0.92 ± 0.09	0.91 ± 0.07	1.272	0.288	
Submaximal function							
6MWT distance (m)	603 ± 112	624 ± 82	689 ± 86	719 ± 90	9.357	** < 0.001**	†*, §***, ¥**
Mental Health							
GAD-7 score	4 (2–7)	5 (2–7)	2 (0–4)	2 (0–3)	12.407^a^	**0.006**	¥*
< 5 none/minimal, n (%)	18 (51)	21 (62)	17 (94)	23 (88)			
≥ 10 moderate, n (%)	3 (9)	3 (9)	0 (0)	1 (4)			
≥ 15 severe, n (%)	1 (3)	2 (6)	0 (0)	0 (0)			
PHQ-9 score	6 (3–10)	8 (5–12)	3 (2–4)	1 (0–3)	40.929^a^	** < 0.001**	§***, #**, ¥***
< 5 none/minimal, n (%)	12 (34)	6 (18)	13 (72)	24 (92)			
≥ 10 moderate, n (%)	10 (29)	6 (18)	1 (6)	1 (4)			
≥ 15 moderate to severe, n (%)	4 (11)	6 (18)	0 (0)	0 (0)			
PCL5 post-trauma stress score	10 (4–24)	9 (6–19)	4 (0–6)	1 (0–5)	25.680^a^	** < 0.001**	§**, #**, ¥***
> 32 PTSD cut-off, n (%)	3 (9)	9 (26)	0 (0)	0 (0)			
Quality of Life: EQ5D	70 (55–80)	69 (40–75)	82 (70–89)	81 (74–90)	21.687^a^	** < 0.001**	†*, §*, #**, ¥**
Fatigue							
FAS	23 (17–29)	26 (22–31)	17 (14–19)	15 (13–17)	41.722^a^	** < 0.001**	†*, §***, #***, ¥***
> 21 cut off –fatigued, *n* (%)	20 (57)	20 (59)	3 (17)	1 (4)			

Bold denotes a statistically significant result, with level indicated by asterisk(s)

Abbreviations: 6MWT, six-minute walk test; GAD-7, general anxiety disorder 7-item checklist, PHQ-9, patient health questionnaire 9 item checklist; PTSD, post-traumatic stress disorder; EQ5D, European Quality of Life 5 domains; FAS, fatigue assessment scale. H, hospitalised illness; CS, community illness with on-going symptoms (community-symptomatic), CR, community illness now recovered (community-recovered; COM, age-, gender- and job-role-matched comparison population. There was no significant difference between CR and COM for any parameter

^†^, H vs. CR; §, H vs. COM; #; CS vs. CR; ¥, CS vs. COM; ¶, H vs. CS. Level of significance: *P < 0.05, **P < 0.01, ***P < 0.001. ^a^Kruskal–Wallis test statistic

### Cardiopulmonary Functional Testing

#### Six-Minute Walk Distance

There was no significant difference in distance walked between community-recovered and comparison groups (689 ± 86 vs. 719 ± 90 m, p > 0.05), nor between hospitalised and community-symptomatic groups (603 ± 112 m vs. 624 ± 82 m, *P* > 0.05) (Table 3). Hospitalised individuals walked 85 m less versus community-recovered (*P* = 0.014) and 116 m less than comparisons (*P* < 0.001). Community-symptomatic individuals were not statistically different to community-recovered or comparisons.

#### Cardiopulmonary Exercise Test (CPET)

There were no differences between hospitalised and community-symptomatic individuals or between community-recovered and comparisons in any CPET variable (Table 4).

**Table 4:** Cardiopulmonary exercise testing (CPET) parameters (mean ± SD)

Variable	H	CS	CR	COM	*F* Score	*P* value	Post hoc comparison
CPET							
V̇O_2_ at Rest (ml/kg/min)	4.8 ± 0.9	4.9 ± 1.0	5.5 ± 1.2	5.5 ± 1.8	2.583	0.057	
V̇O_2_ at VT1 (ml/kg/min)	12.3 ± 1.9	14.5 ± 3.9	17.2 ± 3.0	18.2 ± 5.6	14.665	** < 0.001**	†***, §***, ¥**
V̇O_2_ at Peak (ml/kg/min)	30.5 ± 5.4	34.4 ± 7.2	44.3 ± 7.4	43.9 ± 13.1	17.788	** < 0.001**	†***, §***, #**, ¥***
V̇O_2_ at VT1 (% of predicted peak)	43 ± 7	46 ± 11	49 ± 11	56 ± 17	6.470	** < 0.001**	§***, ¥***
V̇O_2_ at Peak (% of predicted)	108 ± 16	111 ± 19	122 ± 19	133 ± 25	9.510	** < 0.001**	§***, ¥***
Workload at VT1 (W)	70 ± 15	85 ± 33	100 ± 26	109 ± 34	11.036	** < 0.001**	†**, §***, ¥**
Workload at peak (W)	231 ± 35	255 ± 61	308 ± 60	304 ± 65	12.641	** < 0.001**	†***, §***, #**, ¥**
Workload at peak (% of predicted)	97 ± 17	100 ± 23	115 ± 16	127 ± 32	10.692	** < 0.001**	†*, §***, ¥***
W/Kg at VT1	0.74 ± 0.17	0.92 ± 0.36	1.20 ± 0.29	1.38 ± 0.38	25.266	** < 0.001**	†***, §***, #*, ¥**
W/Kg at Peak	2.44 ± 0.47	2.77 ± 0.68	3.73 ± 0.67	3.89 ± 0.82	14.086	** < 0.001**	†***, §***, #***, ¥***
Δ V̇O_2_ (l/min)/Δ Work (W)	10.9 ± 1.0	11.2 ± 2.2	11.2 ± 0.9	11.5 ± 0.7	1.971	0.123	
Lactate at rest (mmol/L)	1.3 ± 0.5	1.3 ± 0.5	1.4 ± 0.4	1.2 ± 0.4	0.691	0.559	
Lactate at peak (mmol/L)	12.1 ± 2.5	13.1 ± 2.3	14.1 ± 2.4	14.2 ± 1.5	5.393	**0.002**	†*, §**
O_2_ Pulse	16.7 ± 3.8	18.5 ± 4.7	21.1 ± 3.7	21.1 ± 4.7	7.131	** < 0.001**	†** §**
O_2_ Pulse (% of predicted peak)	97 ± 20	105 ± 20	119 ± 17	126 ± 22	12.045	** < 0.001**	†*, §***, ¥*
Heart rate profile							
HR at rest (bpm)	82 ± 11	84 ± 13	77 ± 15	73 ± 8	4.791	**0.004**	§*, ¥**
HR at VT1 (bpm)	106 ± 14	108 ± 15	107 ± 12	107 ± 8	0.163	0.921	
HR at peak (bpm)	172 ± 15	175 ± 16	178 ± 7	175 ± 8	0.833	0.479	
% of predicted max HR	110 ± 10	108 ± 9	107 ± 5	108 ± 7	0.650	0.585	
HRR after 1-min (bpm)	25 ± 10	28 ± 11	30 ± 16	26 ± 8	1.053	0.372	
Blood pressure (mmHg)							
Resting systolic BP (mmHg)	126 ± 10	120 ± 11	117 ± 10	121 ± 10	3.670	**0.015**	†*
Resting diastolic BP (mmHg)	85 ± 7	84 ± 8	79 ± 8	79 ± 6	5.795	**0.001**	†*, §**, ¥*
VT1 systolic BP (mmHg)	142 ± 17	144 ± 15	135 ± 17	142 ± 14	1.381	0.252	
VT1 diastolic BP (mmHg)	85 ± 17	83 ± 16	76 ± 9	82 ± 12	1.681	0.175	
Peak systolic BP (mmHg)	169 ± 20	171 ± 18	160 ± 23	166 ± 37	1.392	0.249	
Peak diastolic BP (mmHg)	73 ± 27	79 ± 19	66 ± 20	73 ± 22	1.526	0.212	
Ventilation							
BF at rest (breaths/min)	17 ± 5	16 ± 5	14 ± 3	16 ± 4	0.998	0.397	
BF at VT1 (breaths/min)	20 ± 7	21 ± 6	18 ± 4	20 ± 4	1.158	0.329	
BF at peak (breaths/min)	47 ± 12	47 ± 12	46 ± 6	51 ± 11	1.445	0.234	
V̇E/ V̇CO_2_ at rest	30.8 ± 4.8	30.5 ± 5.3	28.1 ± 2.0	28.0 ± 3.1	3.462	**0.019**	
V̇E/ V̇CO_2_ at VT1	27.8 ± 4.0	26.7 ± 4.0	24.1 ± 1.7	24.3 ± 2.0	7.807	** < 0.001**	†**, §**, ¥*
V̇E/ V̇CO_2_ at peak	34.4 ± 5.5	33.2 ± 4.0	30.5 ± 3.1	31.3 ± 3.3	4.431	**0.006**	†*, §*
V̇E/ V̇CO_2_ slope	29.6 ± 5.1	27.9 ± 5.3	24.1 ± 6.0	25.5 ± 2.6	6.422	** < 0.001**	†**, §*
pCO_2_ rest	5.2 ± 0.8	4.9 ± 0.6	5.1 ± 0.6	5.1 ± 0.5	1.927	0.130	
pCO_2_ peak	4.5 ± 0.9	4.3 ± 0.8	4.6 ± 0.6	4.4 ± 0.6	0.855	0.467	
OUES	3.01 ± 0.58	3.34 ± 0.96	4.88 ± 1.11	3.79 ± 0.93	3.689	**0.014**	†**
Resting spirometry							
FEV1 value (L)	3.7 ± 0.6	3.9 ± 0.8	4.3 ± 0.4	4.1 ± 0.6	2.859	**0.041**	
FEV1% predicted	96 ± 14	93 ± 11	97 ± 14	102 ± 13	2.362	0.076	
FVC value (L)	4.7 ± 0.9	5.2 ± 1.1	5.7 ± 0.6	5.3 ± 0.7	4.751	**0.004**	†**
FVC % predicted	99 ± 14	101 ± 13	106 ± 11	108 ± 10	3.142	**0.028**	§*

Bold denotes a statistically significant result, with the level denoted by asterisk(s)

Abbreviations: VT1, 1^st^ ventilatory threshold; HR, heart rate; HRR, heart rate recovery; BP, blood pressure; BF, breathing frequency; OUES, oxygen uptake efficiency slope. H, hospitalised illness; CS, community illness with on-going symptoms (community-symptomatic), CR, community illness now recovered (community-recovered; COM, age-, gender- and job-role-matched comparison population. There was no significant difference between H versus CS and CR versus COM for any CPET-related parameter

^†^, H vs. CR; §, H vs. COM; #, CS vs. CR; ¥, CS vs. COM. Level of significance: *P < 0.05, **P < 0.01 ***P < 0.001

#### Heart Rate Profile

**Fig. 3:**
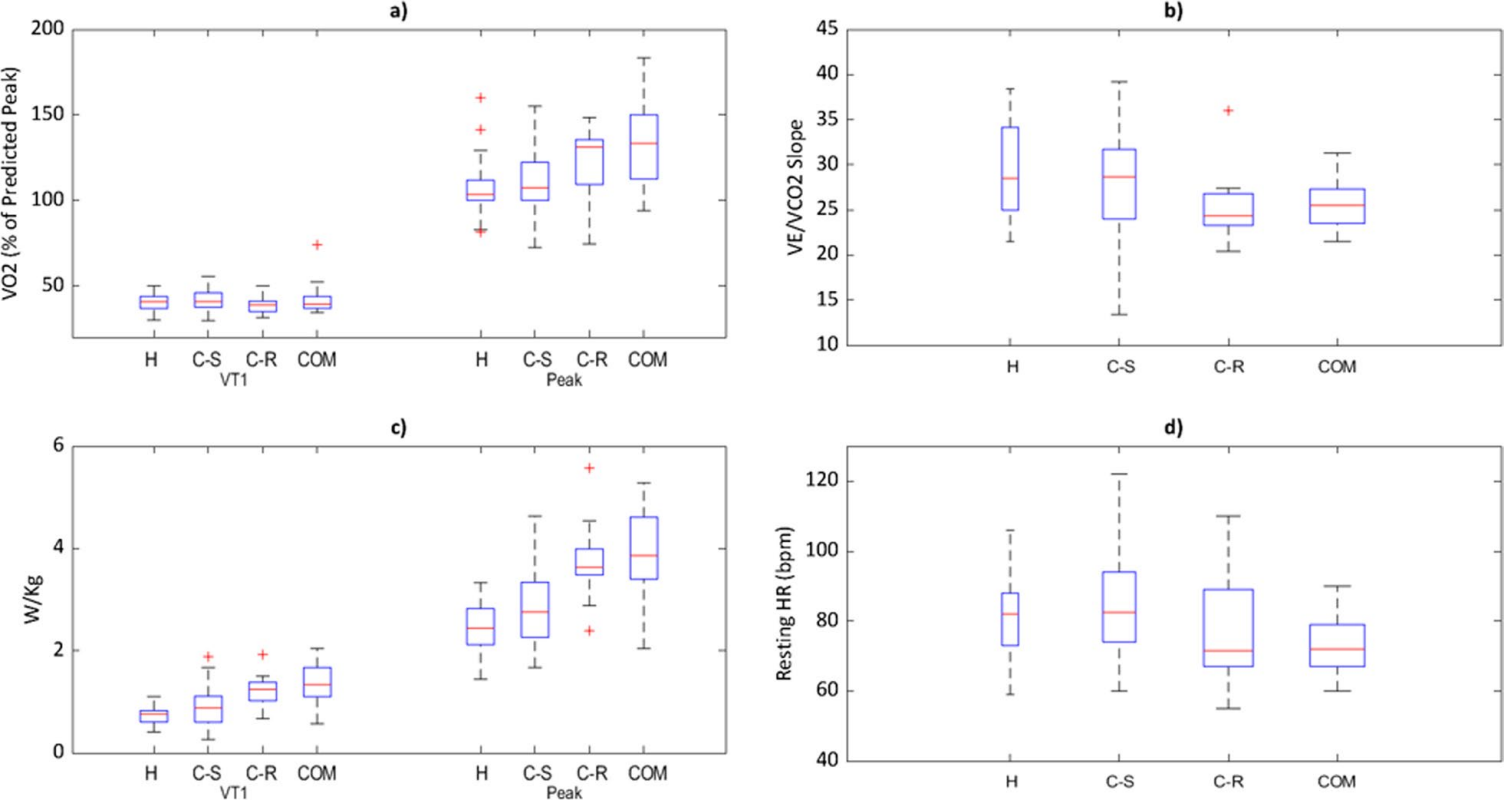
Cardiopulmonary exercise test (CPET) variables: **a** percentage predicted VO2 at VT1 and peak, **b** V̇E/V̇CO2 slope, **c** workload (watts per kilogram) at VT1 and peak, **d** resting heart rate

Hospitalised and community-recovered individuals had a significantly higher resting HR vs comparisons (82 ± 11 bpm and 84 ± 13 bpm vs. 73 ± 8 bpm, both *P* < 0.05) (Table 4, Fig. 3). There were no other between-group differences in exercise HR parameters.

#### Oxygen Uptake

Hospitalised individuals had lower oxygen uptake (V̇O_2_) at VT1 [earlier anaerobic transition] vs community-recovered and comparisons (12.3 ± 1.9 vs 17.2 ± 3.0 and 18.2 ± 5.6 ml/kg/min, both *P* < 0.001). Both the hospitalised and community-recovered groups demonstrate significantly lower values for V̇O_2_ at peak exercise vs comparisons (30.5 ± 5.4 and 34.4 ± 7.2 vs. 43.9 ± 13.1 ml/kg/min, both *P* < 0.001) (Table 5, Fig. 3). Hospitalised and community-symptomatic groups had a reduced mean predicted V̇O_2_ at peak exercise vs community-recovered and comparisons (Table 4).

**Table 5:** Prevalence of participants with abnormal and clinically significant findings following clinical investigations. Descriptive data detailing the total number in each group and percentage based on the number of tests performed

	H	CS	CR	COM
CT				
Tests performed	34	34	18	0
Abnormal result	20 (58%)	2 (6%)	2 (11%)	–
Clinically significant	2 (6%)	0 (0%)	0 (0%)	–
CTPA				
Tests performed	32	34	18	0
Abnormal result	8 (25%)	1 (3%)	0 (0%)	–
Clinically significant	0 (0%)	0 (0%)	0 (0%)	–
CMR				
Tests performed	35	34	18	26
Abnormal result	4 (11%)	5 (15%)	3 (17%)	1 (4%)
Clinically significant	0 (0%)	0 (0%)	0 (0%)	0 (0%)

Abbreviations: CT, computerised tomography; CTPA, computerised tomography pulmonary angiogram; CMR, cardiovascular magnetic resonance imaging. H, hospitalised illness; CS, community illness with on-going symptoms (community-symptomatic), CR, community illness now recovered (community-recovered; COM age-, gender- and job-role-matched comparison population

Hospitalised participants had lower ventilatory efficiency (higher V̇E/V̇CO_2_ slope) than both community-recovered and comparisons (30 ± 5 vs 24 ± 6 and 26 ± 3, both *P* < 0.001) (Table 4, Fig. 3). There were no other significant between-group ventilatory differences.

#### Workload (Watts)

Workloads at VT1 and peak were lower by 36% and 24%, respectively, in hospitalised individuals compared to comparisons (both *P* < 0.001). Workloads at VT1 and peak were lower by 30% and 25%, respectively, in hospitalised versus community-recovered (*P* = 0.002 and *P* < 0.001, respectively). Workloads for VT1 and peak were also less in community-symptomatic vs comparisons by 22% and 16% (*P* = 0.008 and *P* = 0.005, respectively) (Table 4, Fig. 3). No significant between-group differences were reported in RPE or SoB scores during rest, VT1 or peak exercise, or RER at peak.

#### Lung Function Testing

Post hoc analyses revealed no significant between-group differences in FEV1; however, FVC values were significantly lower in hospitalised participants vs community-recovered (4.7 ± 0.9 vs. 5.7 ± 0.6 l, *P* = 0.003) (Table 4). One-way ANOVA revealed a significant between-group difference in % predicted DLCO (H, 83 ± 16%; CS, 91 ± 19%; CR, 90 ± 14%; COM, 98 ± 10%; *F* = 4.132, *P* = 0.008). Post hoc analysis revealed a 15% higher score in % predicted DLCO in comparisons versus hospitalised (*P* = 0.005). No significant between-group differences were reported in % predicted transfer coefficient for carbon monoxide (KCO) (H, 102 ± 19%; CS, 102 ± 12%; CR, 96 ± 11%; COM, 100 ± 7%; *F* = 0.929, *P* = 0.430).

### Blood Testing

There were no between-group differences, aside from white cell count between the hospitalised and community-recovered (6.1 ± 1.3 × 10^9^/l vs. 5.0 ± 1.5 × 10^9^/l) (Additional file 1).

### Body Composition

Hospitalised and community-symptomatic individuals demonstrate the least favourable body composition (Table 3). There were no significant between-group differences in height or waist-to-hip ratio. However, hospitalised and community-symptomatic individuals both had significantly greater body mass index (BMI) values versus community-recovered and comparisons (H, 31 ± 4 kg m^2^; CS, 29 ± 4 kg m^2^; CR, 26 ± 2 kg m^2^; COM, 25 ± 3 kg m^2^). Body mass was greater in hospitalised and community-symptomatic individuals, and reviewing waist circumference scores, this can be attributed to increased abdominal fat (H, 101 ± 13 cm; CS, 96 ± 13 cm; CR, 85 ± 10 cm; COM 86 ± 7 cm). There was no difference in body composition between community-recovered and comparisons.

### Cardiopulmonary Imaging

Imaging results were reviewed by consultants in radiology, cardiology and respiratory medicine to determine clinical significance (Table 5). The only clinically significant pathology identified, moderate volume ground glass changes, occurred on two HRCTs.

### Mental Health and Quality of Life

The mean scores for anxiety and depression equated to ‘minimal’ (0–4) or ‘mild’ (4–9) severity for each group (Table 3). Post hoc analyses revealed a significant difference between community-symptomatic and comparisons for anxiety (*P* = 0.006). Additionally, there were significant differences for depression between hospitalised and community-recovered (*P* < 0.001), hospitalised and comparisons (*P* < 0.001), community-symptomatic and community-recovered (*P* < 0.001) and community-symptomatic and comparisons (*P* < 0.001). The number of hospitalised and community-symptomatic participants scoring ‘none or minimal’ or ‘ ≥ moderate symptoms’ differed vs community-recovered and comparisons (Table 3). Only half of hospitalised individuals reported ‘none or minimal’ anxiety, and one third ‘none or minimal’ depression, vs ~ 90% of comparisons. 29% and 18% of hospitalised and community-symptomatic individuals reported ‘ ≥ moderate depression’ vs 4% of comparisons. PTSD scores were higher in the hospitalised and community-symptomatic vs community-recovered and comparisons (*P* < 0.05). Hospitalised and community-symptomatic participants had lower QoL vs community-recovered and comparisons (*P* < 0.05).

Mean FAS values were significantly higher for hospitalised individuals vs community-recovered (23 [IQR = 17–29] vs. 17 [14–19], *P* = 0.032) and comparisons (15 [10–18], *P* < 0.001) (Table 3). Mean FAS values were also significantly higher in the community-symptomatic (26 [22–31]) versus community-recovered and comparisons (both *P* < 0.001).

### Cognitive Function

There were no between-group differences in fluid, crystallised or total composite scores (Additional file 1).

## Discussion

In a physically active working-age population, this study found that individuals who were symptomatically recovered following community-based acute illness did not differ from an age-, gender- and job-role frequency-matched comparison population across a comprehensive array of cardiopulmonary, functional, neurocognitive and mental health assessments. There were multiple clinically and statistically significant differences between comparisons and those with initially severe illness and ongoing symptomatic illness, including in functional, cardiopulmonary and mental health outcomes.

### Functional Limitations

Hospitalised and community-symptomatic participants had reduced exercise capacity during sub-maximal testing, as seen by shorter distances in the 6MWT, in excess of the minimal clinically significant difference [48], and reduced workload at VT1. The value of sub-maximal testing is that it reflects the ability to perform sustained low-level exercise, including activities of daily living, and therefore may provide an objective insight into an individual's ability to manage with everyday tasks and likelihood of developing fatigue—as seen by half and two-thirds of these groups reporting fatigue as a symptom (Table 2). Other studies [23, 49] have found similar discrepancies in 6MWT, albeit at much shorter distances (reflecting the pre-morbid fitness of participants in this study), with one of those studies repeating the CPET 3 months later [50]. Whilst this showed improvement, but not resolution, of limitations, the inter-visit time interval was short, perhaps not reflecting the time that a full recovery from COVID-19 takes.

There were also limitations seen at maximal exertion (as defined by RER > 1.1) in the same groups (hospitalised and community-recovered), with reduction in absolute and relative V̇O_2_, and workload at both VT1 and peak, with significantly lower peak lactate and O_2_ pulse values. This inability to fully perform is significant for populations who rely on physical performance, preventing a full return to occupational requirements. CPET has been demonstrated to be helpful in identifying limitations and potential causes, including dysfunctional states (such as ventilatory), organ pathology, dysautonomia and deconditioning [6, 51, 52], and the M-COVID study allows us to further investigate some of these potential causes.

Unsurprisingly, given the high prevalence of SoB symptoms (63%), ventilatory inefficiencies were seen in hospitalised individuals, with higher V̇E/V̇CO_2_ slopes compared to the other three groups, a consistent finding for individuals with more initially severe COVID-19 illness [23, 27, 28]. Singh et al*.* [22] also reported reduced V̇O_2_ max with increased V̇E/V̇CO_2_ slopes in individuals recruited from an unexplained exercise intolerance clinic. Possible reasons include ventilation-perfusion mismatch, organ pathology, or hyperventilation, with previous work highlighting the need to correlate both spirometry and diffusion capacity [23, 53] to understand this effect. In this study, lung function results were reassuring, with the only demonstrable effects an 18% reduction in FVC in hospitalised vs. community-recovered, and a 15% reduction in DLCO for hospitalised vs. comparisons. The coincidence of relatively reduced FVC and DLCO in those hospitalised, with no difference in KCO, is suggestive that these differences result from a reduced lung volume, rather than a problem of ventilation-perfusion matching.

Despite concerns regarding end-organ damage after COVID-19 [3, 24, 25, 46, 53–55], especially in athletes [56], this study reassuringly demonstrates an extremely low level of abnormalities in cardiopulmonary imaging, excluding this as a cause for reduced cardiopulmonary functional ability. Hospitalised individuals were more likely to have pathological findings on imaging, however, only 6% were deemed clinically significant (requiring clinical follow up), a much lower rate than the 29–60% previously reported (within methodological differences) (Table 3) [23, 49, 57]. This could be due to the protective effect of cardiorespiratory fitness and lean muscle tissue/metabolic flexibility in this trained population [57, 58].

### Mental Health and Neuro-cognition

There were multiple between-group differences in mental health status, fatigue and QoL. Those in the community-symptomatic group had the highest scores for anxiety, depression and fatigue and the lowest QoL. Those in the hospitalised group scored highest for post-traumatic stress. The clinical significance of this, with higher proportions of moderate and severe symptoms, is seen in Table 3. The impact of the virus can be partitioned using the comparison group, to separate out the impact of social upheaval, isolation, media and other negative effects of the pandemic, including repeated lockdown [59–62]. In particular, for this population, an inability to perform everyday and/or maximal tasks might lead to perceived fear of loss of job, contributing further to the high levels of mental health symptoms. Given the global effect of anxiety, this might also contribute to hyperventilation during CPET, as seen by increased breathing frequencies in the hospitalised and community-symptomatic groups. These findings are similar to those in other study populations [47], and the 2003/4 SARS epidemic [63, 64].

Neurocognitively, the ability to react, analyse and process information (reflected by the ‘fluid composite score’), and acquired knowledge and learning (‘crystallised composite score’), were reviewed. The former is impacted by biological insult, whilst the latter is relatively preserved. Despite work in a similar population displaying significant changes,(30) our findings suggest no medium-term damage, with deficits most evident in the community-symptomatic group and no statistically significant differences seen. Previous work has demonstrated significant improvement with time [8, 66].

### Participant Demographics

There were no between-group differences in highest educational attainment or rank, as proxies for SES (Additional file 1), but significant between-group differences were demonstrated in age and body composition (*P* > 0.05). Hospitalised individuals were older than community based groups, and both hospitalised and community-symptomatic individuals had increased body mass, BMI and waist circumference vs community-recovered, consistent with increased age and BMI as risk factors for COVID-19 severity [9, 46, 47]. These demographic differences may have influenced study outcomes. However, given all military personnel are required to meet the same fitness standards, including the comparison group, and relative CPET measurements are age and weight calculated, this effect should be mitigated.

### Strengths and Limitations

This is the first study, to our knowledge, that has compared groups, across the spectrum of acute COVID-19 severity, including on-going or resolved symptom cohorts, with an age-, gender- and job-role frequency-matched comparison group, to identify ongoing organ pathology, functional limitations and mental health impact in a young, working-age population required to undertake high levels of physical activity. Whilst the sample size (*n* = 113) is modest, this is balanced by the comprehensive assessment completed in every participant.

An additional strength is the population studied. Although having a predominantly male, younger population might be a risk of participant bias, this tightly-defined and generally healthy population reduce confounders and allow the effect of COVID-19 to be seen. Whilst not all findings can be extrapolated to the wider population, which is a limitation, the impact on COVID-19 on sportspeople and other physically demanding occupations has been a research priority [70]. Steps were taken to minimise selection bias during recruitment, with consecutive eligible participants approached until the study was filled. Initial sample size calculations were unable to be performed in Summer 2020 due to the unknown quality of COVID-19, therefore no power calculations are possible. Throughout this study, all investigations were delivered by the same team of investigators, equipment and conditions, increasing the consistency of the data.

There are limitations to this study. A key limitation is that of the differences between age and BMI between the groups, which might have independently impacted on the cardiopulmonary and functional outcomes, as well as increasing the risk of initial severe and worse prognosis. Armed Forces fitness standards should be met by all individuals, and CPET measurements are age and weight calculated, so it is hoped that might mitigate the effect. A further limitation is lack of pre-COVID-19 participant data, which prevents the partitioning of effect pre- and post-disease.

## Conclusion

This study showed that those with more severe acute disease and/or prolonged symptoms were older and had a higher BMI. Within these groups, there is an increased likelihood of pathological cardiopulmonary imaging findings (albeit at a much lower rate than other published studies) and reduced exercise capacity during sub-maximal and maximal testing. These same groups also experienced higher rates of mental health symptoms, fatigue, and a reduced QoL. The most common symptoms (Table 2) are reflective of those in other studies, which supports the generalisability of other findings here, such as objective cardiopulmonary fitness and neurocognitive outcomes, which have not previously been reported in case-controlled cohorts [47, 67–69].

Reassuringly, this study also found that recovered community-based individuals do not differ from a matched comparison population in any parameter, which will reassure the majority of recovered individuals with less severe disease, and the clinicians responsible for their care. It will permit the dedication of resources to those who remain at risk of important clinical sequelae, as our findings suggest that for individuals who will be exposed to high intensity physical exercise, who were either hospitalised during acute illness or experience prolonged symptoms, that a specific, comprehensive evaluation of functional and neurocognitive capacity, mental health status and cardiopulmonary pathology is warranted [29, 71, 72].

## Supplementary Information

**Additional file 1.** Education, rank, cognitive, and blood test results for the MCOVID participants.

## Data Availability

Data relate to the serving population of the Ministry of Defence and thus are sensitive. Research teams requesting data are invited to contact the corresponding author and appropriate permissions will be sought for release.
