# Supplementary material for: Cardiopulmonary, Functional, Cognitive and Mental Health Outcomes Post-COVID-19, Across the Range of Severity of Acute Illness, in a Physically Active, Working-Age Population
Source: Sports Med Open. 2023 Feb 2;9:7. doi: 10.1186/s40798-023-00552-0 (PMC9893959; doi:10.1186/s40798-023-00552-0)
Supplement: Supplementary file 1 — Additional file 1. Education, rank, cognitive, and blood test results for the MCOVID participants. [file 40798_2023_552_MOESM1_ESM.docx]

**Supplementary File 1. Education, rank, cognitive, and blood test results for the MCOVID participants**

**Title:** Cardiopulmonary, functional, cognitive and mental health outcomes post COVID-19, across the range of severity of acute illness, in a physically active working age population

**Journal:** Sports Medicine Open

**Authors:** *Oliver O’Sullivan^a,b^ , *David A Holdsworth^c,d^, Peter Ladlow^a,e^, Robert M Barker-Davies^a,f^, Rebecca Chamley^c,d^, Andrew Houston^a^, Samantha May^a^, Dominic Dewson^a^, Daniel Mills^a^, Kayleigh Pierce^d,g^, James Mitchell^a,h^ Cheng Xie^d^, Edward Sellon^d^, Jon Naylor^g^, Joseph Mulae^g^, Mark Cranley^i^, Nick P Talbot^d,j^, Oliver J Rider^k,l^ , Edward D Nicol^c,m^ and Alexander N Bennett^a,n^ *joint-first authors

**Affiliations:**

^a^ Academic Department of Military Rehabilitation (ADMR), Defence Medical Rehabilitation Centre (DMRC) Stanford Hall, Loughborough, UK.

^b^ Academic Unit of Injury, Recovery and Inflammation Sciences, University of Nottingham, Nottingham, UK

^c^ Academic Department of Military Medicine, Birmingham, UK.

^d^ Oxford University Hospitals NHS Foundation Trust, Oxford, UK

^e^ Department for Health, University of Bath, Bath, UK

^f^ School of Sport, Exercise and Health Sciences, Loughborough University, Loughborough, UK

^g^ Royal Centre for Defence Medicine, Birmingham, UK

^h^ Metabolic Neurology, Institute of Metabolism and Systems Research, University of Birmingham, Birmingham, UK

^i^ Defence Medical Rehabilitation Centre (DMRC), Stanford Hall, Loughborough, UK.

^j^ Department of Physiology, Anatomy and Genetics, University of Oxford, Oxford, UK

^k^ University of Oxford Centre for Clinical Magnetic Resonance Research, University of Oxford, Oxford, UK

^l^ Department of Cardiology, Oxford University Hospitals NHS Foundation Trust, Oxford, UK

^m^ Royal Brompton Hospital, London, UK

^n^ National Heart and Lung Institute, Imperial College London, London, UK

**Corresponding author:** Prof Alexander Bennett, Defence Medical Rehabilitation Centre (DMRC) Stanford Hall, Stanford on Sour, Loughborough, LE12 5QW. Email: [Alexander.N.Bennett@btinternet.com](mailto:Alexander.N.Bennett@btinternet.com)

**Table 1:** Descriptive data detailing the number (and percentage) of educational attainment and current military rank in each COVID19 severity group.

|  | **H** | **CS** | **CR** | **COM** |
| --- | --- | --- | --- | --- |
| **Education** | | | | |
| GCSE (or equiv.) | 14 (40%) | 7 (21%) | 4 (22%) | 5 (19%) |
| A-Level (or equiv.) | 14 (40%) | 10 (29%) | 4 (22%) | 7 (27%) |
| Bachelors | 5 (14%) | 11 (32%) | 7 (39%) | 11 (42%) |
| > Bachelors | 2 (6%) | 6 (18%) | 3 (17% | 3 (12%) |
| **Rank** | | | | |
| Junior NCO’s | 9 (26%) | 14 (41%) | 4 (22%) | 2 (8%) |
| Senior NCO | 17 (49%) | 10 (29%) | 3 (17%) | 12 (46%) |
| Officer Ranks | 9 (26%) | 10 (29%) | 11 (61%) | 12 (46%) |

Abbreviations: GCSE, General Certificate of Secondary Education; NCO, non-commissioned officer, H, hospitalised illness; CS, community illness with on-going symptoms (community-symptomatic), CR, community illness now recovered (community-recovered; COM age, gender and job-role matched comparison population.

No association between educational level and COVID19 severity was demonstrated between groups (χ2 = 13.352, p=0.147). An association was found between rank and COVID19 severity (χ2 = 16.045, p=0.014), however the trends do not align with any particular narrative (Table 1).

**Table 2:** Cognitive Functioning

|  | **H** | **CS** | **CR** | **COM** |
| --- | --- | --- | --- | --- |
| Fluid Composite | 54 ± 11 | 50 ± 12 | 55 ± 17 | 57 ± 15 |
| Crystalised Composite | 55 ± 11 | 58 ± 10 | 54 ± 15 | 55 ± 14 |
| Total Composite | 55 ± 11 | 55 ± 10 | 55 ± 16 | 57 ± 15 |

Abbreviation: H, hospitalised illness; CS, community illness with on-going symptoms (community-symptomatic), CR, community illness now recovered (community-recovered; COM age, gender and job-role matched comparison population.

A one-way ANOVA revealed no significant differences between groups in fluid composite (p=0.147), crystalised composite (p=0.366) and total composite (p=0.863) (Table 2).

**Table 3:** Blood Markers

| **Variable** | **H** | **CS** | **CR** | **COM** | **F Score** | **P Value** | **Post-Hoc Comparison** |
| --- | --- | --- | --- | --- | --- | --- | --- |
| Haemoglobin (g/L) | 152 ± 9 | 150 ± 13 | 150 ± 8 | 150 ± 12 | 0.129 | 0.943 |  |
| White Blood Cell Count (K/uL) | 6.1 ± 1.3 | 5.5 ± 1.6 | 5.0 ± 0.9 | 5.2 ± 1.3 | 3.518 | **0.018** | †* |
| Platelets (K/uL) | 240 ± 67 | 245 ± 51 | 227 ± 39 | 233 ± 58 | 0.424 | 0.736 |  |
| Sodium (mmol/L) | 140 ± 2 | 136 ± 24 | 141 ± 2 | 141 ± 2 | 0.940 | 0.424 |  |
| Potassium (mmol/L) | 4.0 ± 0.4 | 4.0 ± 0.5 | 4.1 ± 0.4 | 3.9 ± 0.3 | 0.404 | 0.750 |  |
| Urea (mmol/L) | 5.4 ± 1.4 | 5.5 ± 1.3 | 5.2 ± 1.2 | 5.5 ± 1.3 | 0.204 | 0.894 |  |
| Creatinine (μmol/L) | 83 ± 17 | 83 ± 14 | 83 ± 11 | 87 ± 18 | 0.411 | 0.745 |  |
| Alkaline Phosphatase (U/L) | 68 ± 23 | 67 ± 21 | 63 ± 19 | 59 ± 15 | 1.336 | 0.266 |  |
| Albumin (g/L) | 44 ± 2 | 44 ± 4 | 45 ± 2 | 45 ± 2 | 1.783 | 0.155 |  |
| Neutrophils (x 10*9/L) | 3.3 ± 1.0 | 3.1 ± 1.4 | 2.5 ± 0.9 | 2.8 ± 0.8 | 2.140 | 0.099 |  |
| Lymphocytes (x 10*9/L) | 2.1 ± 0.6 | 8.0 ± 35.9 | 1.9 ± 0.4 | 1.8 ± 0.5 | 0.725 | 0.539 |  |
| Eosinophils (x 10*9/L | 0.2 ± 0.2 | 0.2 ± 0.1 | 0.2 ± 0.1 | 0.2 ± 0.1 | 0.183 | 0.908 |  |
| HbA1c (mmol/mol) | 38 ± 7 | 36 ± 5 | 34 ± 3 | 34 ± 3 | 3.832 | **0.012** | §* |
| Thyroid Stimulating Hormone (mU/L) | 2.6 ± 2.3 | 2.7 ± 3.4 | 3.1 ± 3.4 | 2.3 ± 2.9 | 0.275 | 0.843 |  |
| Creatine Kinase (U/L) | 260 ± 521 | 216 ± 390 | 144 ± 68 | 404 ± 664 | 1.217 | 0.307 |  |
| Vitamin D (ng/mL) | 49 ± 14 | 55 ± 25 | 62 ± 30 | 69 ± 34 | 3.307 | **0.023** |  |
| Serum Iron (μmol/L) | 22 ± 7 | 23 ± 6 | 21 ± 7 | 20 ± 7 | 1.027 | 0.384 |  |
| Serum Ferritin (ng/mL) | 213 ± 181 | 149 ± 143 | 121 ± 63 | 126 ± 107 | 2.695 | **0.049** |  |

Abbreviation: H, hospitalised illness; CS, community illness with on-going symptoms (community-symptomatic), CR, community illness now recovered (community-recovered; COM age, gender and job-role matched comparison population.

†, H vs. CR; §, H vs. COM. Level of significance: * p<0.05, ***p<0.001
